# GPCR Inhibition in Treating Lymphoma

**DOI:** 10.1021/acsmedchemlett.1c00600

**Published:** 2022-02-15

**Authors:** Marilia Barreca, Virginia Spanò, Maria V. Raimondi, Roberta Bivacqua, Stefano Giuffrida, Alessandra Montalbano, Andrea Cavalli, Francesco Bertoni, Paola Barraja

**Affiliations:** † Department of Biological, Chemical and Pharmaceutical Sciences and Technologies (STEBICEF), University of Palermo, Via Archirafi 32, 90123 Palermo, Italy; ‡ Institute for Research in Biomedicine, Faculty of Biomedical Sciences, USI, Via Francesco Chiesa 5, 6500 Bellinzona, Switzerland; § Swiss Institute of Bioinformatics, Quartier Sorge - Batiment Amphipole, 1015 Lausanne, Switzerland; ∥ Institute of Oncology Research, Faculty of Biomedical Sciences, USI, Via Francesco Chiesa 5, 6500 Bellinzona, Switzerland; ⊥ Oncology Institute of Southern Switzerland, Via Vincenzo Vela 6, 6500 Bellinzona, Switzerland

**Keywords:** G protein-coupled receptors, GPCRs, lymphoma, MCL, DLBCL, CXCR4

## Abstract

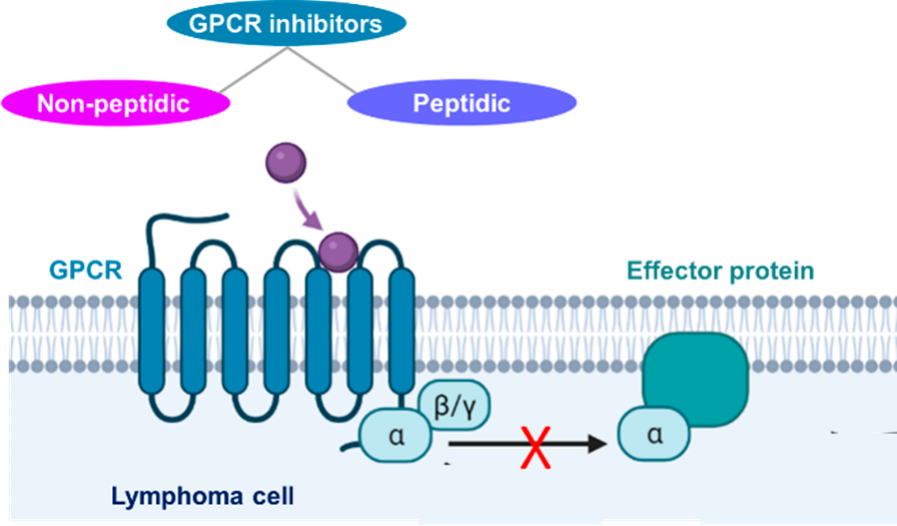

G protein-coupled receptors (GPCRs) are important classes of cell surface receptors involved in multiple physiological functions. Aberrant expression, upregulation, and mutation of GPCR signaling pathways are frequent in many types of cancers, promoting hyperproliferation, angiogenesis, and metastasis. Recent studies showed that alterations of GPCRs are involved in different lymphoma types. Herein, we review the synthetic strategies to obtain GPCR inhibitors, focusing on CXCR4 inhibitors which represent most of the GPCR inhibitors available in the market or under preclinical investigations for these diseases.

G protein-coupled receptors (GPCRs) are the largest family of integral membrane proteins in the human genome and mediate the majority of cellular responses to endogenous (amines, cations, lipids, peptides and glycoproteins) and exogenous (therapeutic drugs, light, tastants, and odorants) ligands and stimuli. Based on sequence homology and functional similarity, they are classified into six classes: class A, rhodopsin-like receptors; class B,secretin receptors family; class C, metabotropic glutamate receptors; class D, fungal mating pheromone receptors; class E, cAMP receptors; and class F, frizzled (FZD) and smoothened (SMO) receptors.^1^ Despite the diversity of physiological responses, all GPCR members share a common architecture, with an extracellular N-terminus and a cytosolic C-terminus separated by seven transmembrane α-helices connected by three intracellular and three extracellular peptide loops. Activation of GPCRs by ligands induces conformational changes of the receptor, promoting the coupling with heterotrimeric G-protein (Gα, Gβ, and Gγ). After the exchange of GDP for GTP on the Gα subunit, GTP-bound Gα dissociates from Gβγ, and Gα and Gβγ separately modulate downstream signaling cascades. The Gα protein subunit targets adenylyl cyclase, phospholipase C (PLC), cyclic GMP phosphodiesterase, and RhoGTPase nucleotide exchange factors (RhoGEF). The dissociated Gβγ subunit activates other downstream effectors such as ion channels. Besides G-protein, GPCRs can also mediate signal through β-arrestins, multifunctional adaptor proteins that activate several signaling molecules such as c-Src, extracellular regulated kinase (ERK), Janus-activated kinase (JNK), and small GTPase RhoA by forming a complex with them. Some GPCR ligands activate either G-protein or β-arrestin; this event is called as “biased activation”.^2^

Due to the key roles of GPCRs in cell physiology and homeostasis, altered signaling pathways associated with GPCRs are implicated in the pathophysiology of various diseases, including cancer, infections, and metabolic, immunological, or neurodegenerative disorders. Approximately 40% of clinically approved drugs mediate their effects by modulating GPCRs, which makes them attractive targets for drug screening and discovery.^3^

Aberrations such as overexpression, deletion, and mutation of GPCRs have been identified as possible triggering events in lymphoma.

Lymphomas are among the 10 most common cancers, and although progress has been achieved in increasing survival, there is still an unmet need of efficacious approaches. Derived from the transformation of lymphocytes, lymphomas comprise a very diverse series of individual diseases, characterized by specific molecular, biologic, and clinical features.^4^ The most common type is diffuse large B-cell lymphoma (DLBCL), an heterogeneous aggressive tumor containing at least two major subtypes: activated B-cell like (ABC) DLBCL and germinal center B-cell (GCB) type DLBCL, which can be further divided in genetically defined clusters. Other frequent lymphomas include follicular lymphoma (FL), mantle cell lymphoma (MCL), and marginal zone lymphoma (MZL). FL is the most common indolent subtype and the second most common lymphoma. FL is usually incurable, slow-growing, and responsive to initial therapy with disease-free intervals alternating with progressions and relapses. MCL is characterized by an aggressive clinical course typical of aggressive lymphomas plus the incurability with conventional chemotherapy seen in indolent lymphomas. MZLs are indolent lymphomas, and they comprise three distinct diseases (extranodal MZL, splenic MZL, and nodal MZL).

Sequencing studies have revealed mutations of GPCRs in many NHL subtypes. In particular, expression and functional alterations of cannabinoid receptors (CNR1 and CNR2), purinergic receptor P2RY11, chemokine receptors (CXCR3, CXCR4, and CXCR5), sphingosine-1-phospate receptors (S1PR1, S1PR2, and S1PR3), purinergic receptor GPR34, or estrogen receptor 1 (GPER1) have been reported in MCL, FL, DLBCL, and MZL.^5^

Here, we provide an overview of the chemistry behind currently available GPCR inhibitors, focusing on CXCR4 inhibitors. Indeed, most of the inhibitors available in the market or under preclinical investigations specifically target CXCR4. To date, their main application has been to facilitate the collection of hematopoietic stem cells for autologous transplantation. Indeed, due to CXCR4 expression on CD34^+^ hematopoietic stem cells, blocking the binding between CXCR4 and its ligand SDF-1alpha leads to the mobilization of progenitor cells in the peripheral blood. Moreover, CXCR4 is also expressed on tumor cells, including lymphoma cells, and it is a key receptor for metastatic spread, neoplastic cells survival and tumor angiogenesis.^6^ Of interest, CXCR4 is upregulated by lymphoma cells exposed to PI3K, BTK, and SYK inhibitors, and it can contribute to the lymphoma resistance to these agents.^7−9^

From a structural point of view, the GPCR inhibitors (Table 1) have been divided into nonpeptidic (Figure 1) and peptidic compounds, highlighting their different scaffolds.

**Table 1:** Overview of GPCR Inhibitors^a^

international nonproprietary name	development codes	clinical stage^b^	orphan drug status^b^	ongoing trials
nonpeptidic inhibitors				
plerixafor	Mozobil, AMD3100, JM 3100, LM-3100, SDZ SID 791	FDA approved (stem cell mobilization)	stem cell mobilization	yes
mavorixafor	X4P-001, AMD11070, AMD070, ABSK-081	Phase 3	WHIM syndrome	yes
	GENZ-644494, AMD3465	preclinical	no	no
	IQS-01.01RS	preclinical	no	no
	WK1	preclinical	no	no
peptidic inhibitors				
balixafortid	BTK140	preclinical	no	no
	POL6326	Phase 3	no	yes
	LY2510924, T-134	Phase 2	no	no

a WHIM, warts, hypogammaglobulinemia, infections, myelokathexis.

b Based on https://adisinsight.springer.com/ and/or https://clinicaltrials.gov accessed in December 2021.

**Figure 1:**
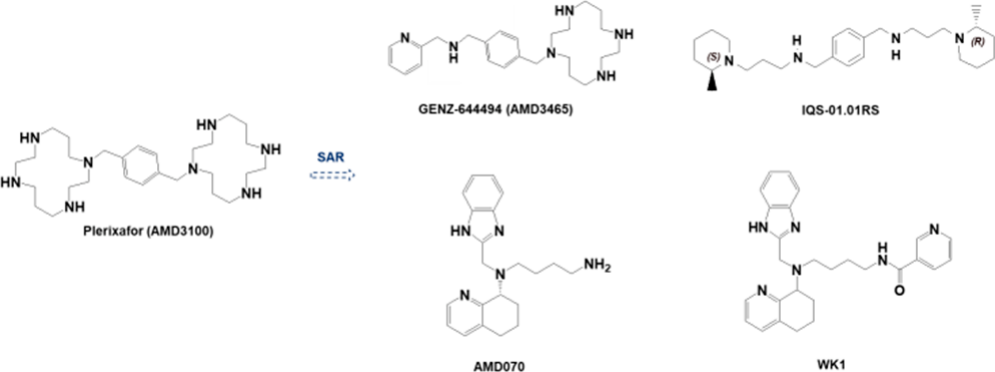
Structures of nonpeptidic GPCR inhibitors.

The most advanced selective CXCR4 antagonist is plerixafor (AMD3100) (Figure 1), a polyamine composed of two monocyclam (1,4,8,11-tetraazacyclotetradecane) rings connected by a *para*-xylylene linker.^10^ Its synthesis was described for the first time in 1987 by Ciampolini et al., but, unfortunately, they did not report the final yield of the product, making it difficult to assess the total synthesis efficiency.^11^

A more convenient synthetic strategy was then proposed to avoid the formation of mono-, di-, or tetrasubstituted cyclam byproducts. Starting from the 1,4,8,11-tetraazacyclotetradecane **1**, the treatment with Cr(CO)_6_ as the protective group led to tridentate complex **2a** which was then selectively alkylated with *para*-xylylene at the unprotected nitrogen atom in the presence of Na_2_CO_3_ and DMF as the solvent. Subsequent removal of the protective groups in HCl led to the formation of plerixafor in high yield (Scheme 1)^12^

**Scheme 1:**
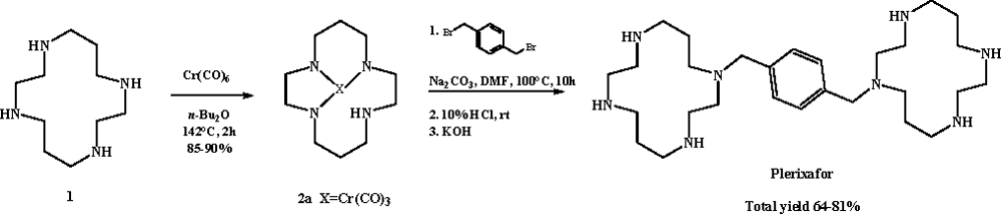
Synthetic Route for Plerixafor Using Cr(CO)_6_

Alternatively, due to the carcinogenicity of Cr(CO)_6_, the use of P(NMe_2_)_3_^13^ or B(NMe_2_)_3_^14^ as the protective group was proposed by Handel et al., giving boron- or phosphoryl-protected cyclams **2b**,**c**,^15^ whose reaction with *para*-xylylene dibromide and acid deprotonation in EtOH formed the target bicyclam with 90% total yield (Scheme 2).

**Scheme 2:**
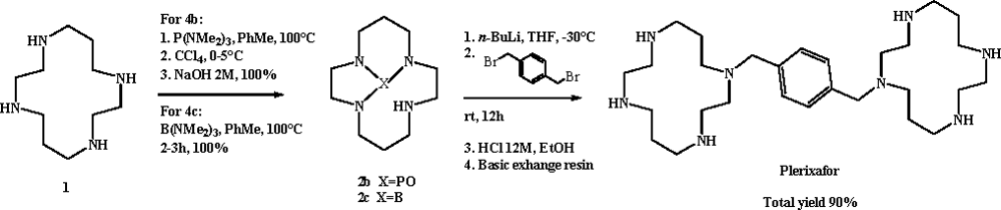
Synthetic Route for Plerixafor Using P(NMe_2_)_3_ or B(NMe_2_)_3_

Plerixafor was approved in 2008 by the U.S. Food and Drug Administration (FDA) for the autologous transplantation of bone marrow (BM) cells in patients with NHL or multiple myeloma. Since then, further *in vitro* and *in vivo* studies have also shown its direct antitumor activity when given in combination with the anti-CD20 monoclonal antibody rituximab.^16^ The *in vitro* inhibition of CXCR4 induced by plerixafor in human Raji and B104 DLBCL-lymphoma cell lines led to the suppression of tumor-promoting signals delivered by the CXCR4/CXCL12 axis. Furthermore, the *in vitro* concomitant administration of plerixafor and rituximab resulted in a dose-dependent decrease of proliferation in both Raji and B104 tumor cells as well as in a significant increase in the survival of Raji tumor-bearing mice. Compared to rituximab alone, the combination treatment (rituximab 10 mg/kg, twice per week plus plerixafor 1 mg/kg, three times per week) significantly extended the median survival, suggesting a noteworthy clinical effect of combining the two drugs.^17^

The synergistic anticancer activity between monoclonal antibody treatment and CXCR4 antagonism has also been confirmed using GENZ-644494 (AMD3465) (Figure 1), a *N*-pyridinylmethylene monocyclam CXCR4 antagonist with inhibitory effect and CXCR4 binding affinity similar to that of plerixafor.^18^ Different from the case of plerixafor, GENZ-644494 has only one cyclam ring linked to an aminomethylpyridine moiety, suggesting that the presence of a single cyclam ring is enough to confer CXCR4 inhibition. Molecular modeling studies of plerixafor and GENZ-644494 have shown that the binding between one cyclam ring and the CXCR4 receptor depends on three positively charged amino acid residues in transmembrane regions (Asp^171^, Asp^262^, and Glu^288^).^19^ In particular, one cyclam ring binds the pocked at Asp^171^ in TM-IV, while the other portion (the second cyclam for plerixafor or the *N*-pyridinylmethylene moiety for GENZ-644494) interacts with the carboxylic acid groups of Asp^262^ and Glu^288^ from TM-VI and -VII, respectively.^20,21^

GENZ-644494 is synthesized via a four-step reaction sequence, starting from the same cyclam used for plerixafor. In the first step, protective groups are introduced at the three nitrogen atoms of compound **1** by reaction with di-*tert*-butyl dicarbonate in DCM. Then, the alkylation of triprotected cyclam **3** with *para*-xylylene and subsequent reaction with 2-(aminomethyl)pyridine in CH_3_CN led to the tetraazamacrocycle **5**. The deprotection in acidic conditions completes the synthesis of GENZ-644494 in 89% yield (Scheme 3).

**Scheme 3:**
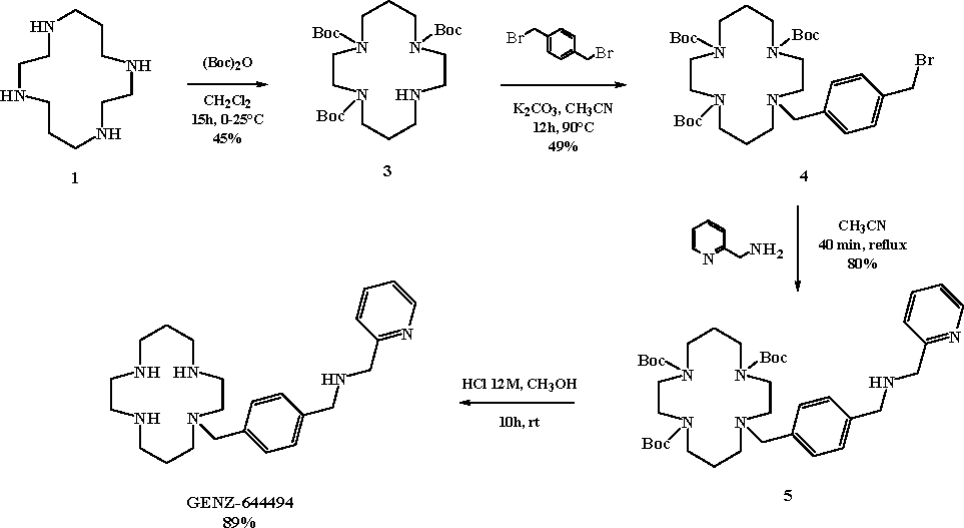
Synthetic Route for GENZ-644494

In analogy to plerixafor, the combination of GENZ-644494 with alemtuzumab and rituximab in *in vivo* Raji and B104 disseminated lymphoma models enhanced the therapeutic efficacy of the single monoclonal antibody. Compared to the vehicle treated control group, mice showed an overall increased survival by 40%. Mechanisms of action that seem to contribute to this activity are the mobilization of tumor cells away from the stroma, the increasing of their vulnerability to the action of monoclonal antibody, and the recruitment of neutrophils mediating antibody-dependent cell-mediated cytotoxicity (ADCC).^17^

The encouraging results obtained with plerixafor boosted further structure–activity relationship (SAR) investigations. With the aim of improving pharmacokinetic properties and overcoming low oral bioavailability,^22^ one or both bicyclams were replaced by heterocyclic rings.

As a result of this lead optimization process, the tetrahydroquinoline derivative AMD070 (mavorixafor) (Figure 1) emerged for its ability to specifically antagonize CXCR4 at the nanomolar level (IC_50_ = 13 nM) in a CXCR4 125I-SDF inhibition binding assay.^23^

The synthetic approach started from intermediate **6** which was subjected to N-alkylation with *N-tert*-butoxycarbonyl-2-chloromethylbenzimidazole, thus leading to derivative **7**. Subsequent reaction with 4-bromovaleronitrile followed by reduction with nickel Raney under H_2_ led to the desired AMD070 as a racemic mixture (Scheme 4), which upon HPLC purification yielded the most active (*S*)-enantiomer.

**Scheme 4:**
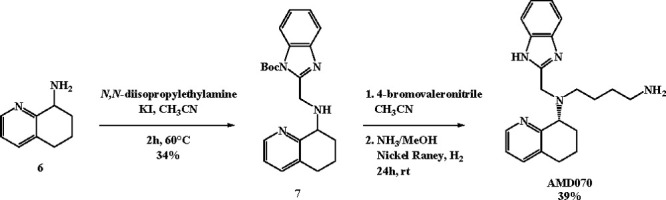
Synthetic Route for AMD070

*In vitro* CXCR4 antagonistic activity exerted by AMD070 was investigated along with the effect of WK1, a niacin derivative of AMD070, synthesized by the same authors according to Scheme 5.^24^

**Scheme 5:**
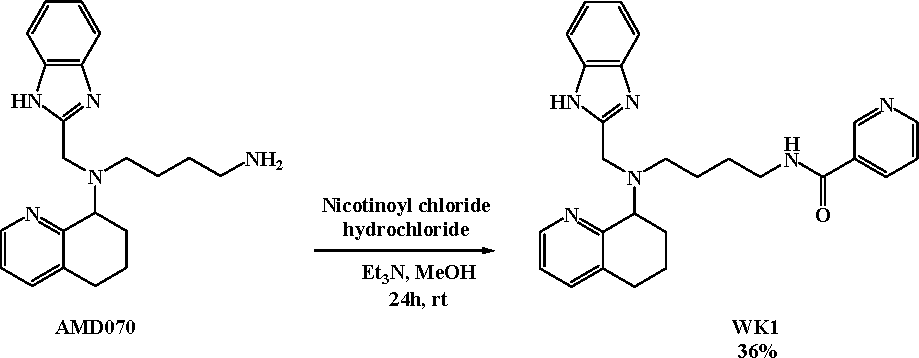
Synthetic Route for WK1

BL2 (Burkitt lymphoma), RI-1 and U2932 (NGCB-DLBCL cell lines), and SU-DHL-4 (GCB-DLBCL cell lines), characterized by the surface expression of CXCR4, were treated with both compounds, thus proving the ability of WK1 to inhibit BL2 and SU-DHL-4 cell growth at IC_50_ values of 15.4 and 26.76 μM, respectively, as well as that of AMD070 at IC_50_ values of 31.18 and 26.76 μM. Compared to plerixafor, WK1 showed more pronounced proapoptotic effects coupled with higher level of cleaved caspase-3 and induction of BCL2 proapoptotic genes.

Although plerixafor demonstrated an excellent ability to counteract B-cell tumor spread in animal models, its cardiotoxicity limits its use. IQS-01.01RS (Figure 1), a new CXCR4 inhibitor recently disclosed, is a noncyclam tetraamine derivative endowed with lower cardiotoxicity and better pharmacodynamic properties than AMD3100. It was obtained through a multistep asymmetric synthesis, starting from 4-(diethoxymethyl)benzaldehyde **9** which was reacted with 1 equiv of 3-[(2*S*)-2-methylpiperidin-1-yl]propan-1-amine **8**(*S*) in the presence of NaBH_4_, thus leading to the corresponding compound 12(*S*). Subsequent hydrolysis resulted in the isolation of 4-[({3-[(2*S*)-2-methylpiperidin-1-yl]propyl}amino)methyl]benzaldehyde **11**(*S*) in 80% yield, which was subjected to reductive amination yielding the tetraamine IQS-01.01RS in 83% yields (Scheme 6).^25^

**Scheme 6:**
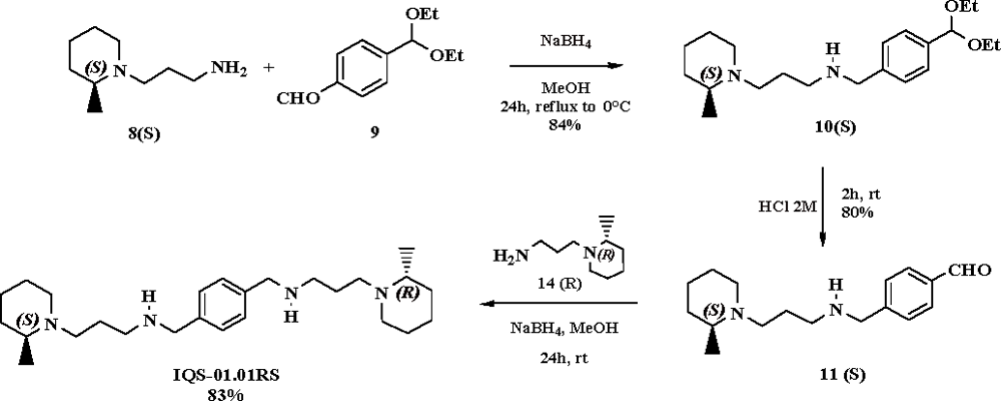
Synthetic Route for IQS-01.01RS

Computational studies indicated that IQS-01.01RS binds to a CXCR4 domain different from that of plerixafor, acting as an allosteric inhibitor and allowing a longer lasting inhibition of the CXCR4/CXCL12 pathway. Indeed IQS-01.01RS induced a 181% inhibition of CXCR4 receptor activity by acting as its inverse agonist.^26^

After 48 h treatment, IQS-01.01RS produced a 40% antiproliferative effect on a panel of 13 GCB/ABC-DLBCL cell lines, compared to 12% induced by plerixafor. In addition, unlike plerixafor, it is able to induce apoptosis in CD19^+^ tumor B cells, interfering with CXCL12-induced migration by acting as a potent inhibitor of cell chemotaxis. Western blot analysis of CXCR4 downstream signaling in SUDHL6 and U2932 cells showed the ability of IQS-01.01RS to inhibit basal and CXCL12-induced phosphorylation of ERK1/2 and AKT, and strong downregulation of the MYC proto-oncogene in ABC- and GCB-DLBCL cells. *In vivo* evaluation in NSG mice showed that the drug combination with CPI203, a BET bromodomain antagonist, induced a decrease in tumor mass of 38%, while as single compounds the reduction was 27% with CPI203 and 45% with IQS-01.01RS, underlining the ability of IQS-01.01RS as a synergizing agent.^26^

The critical role of GPCRs in lymphoma and other neoplastic malignancies has triggered the development of selective peptidic inhibitors for therapeutic use.

BTK140 (or 4F-benzoyl-TN14003) (Figure 2) is a 14-residue bio stable synthetic peptide, derived from a naturally occurring horseshoe crab protein, that not only bind to CXCR4 with higher affinity than plerixafor (4 vs 84 nmol/L) but also dissociate from it with slow fashion. Differently from plerixafor and all other CXCR4 inhibitors that have a rapid reversible bond, this unique ability of BTK140 induce a stronger effect.^27^ BTK140 showed *in vitro* antiproliferative activity in ten cell lines of either germinal center B-cell like (GCB) DLBCL (DBr, DOHH2, SU-DHL-4, CJ, McA, OCI-LY19) and activated B-cell–like (ABC) DLBCL (OCI-LY3, WP, LR, and OCI-LY10), with IC_50_ values ranging from 16.55 to 79.33 nM. The alteration of growth was particularly marked in cells expressing high CXCR4 mRNA and was caused by the inhibition of CXCR4-mediated cell adhesion and migration.^28^ Also in this case, the combinatorial regimen gave satisfactory results. The combination of BTK140 with rituximab further enhanced the apoptotic effect against lymphoma cells, reducing the number of viable cells in the bone marrow up to 93%. Moreover, *in vivo* evaluation in xenograft models of localized and disseminated NHL with bone marrow involvement inhibited the local tumor progression.^29^

**Figure 2:**
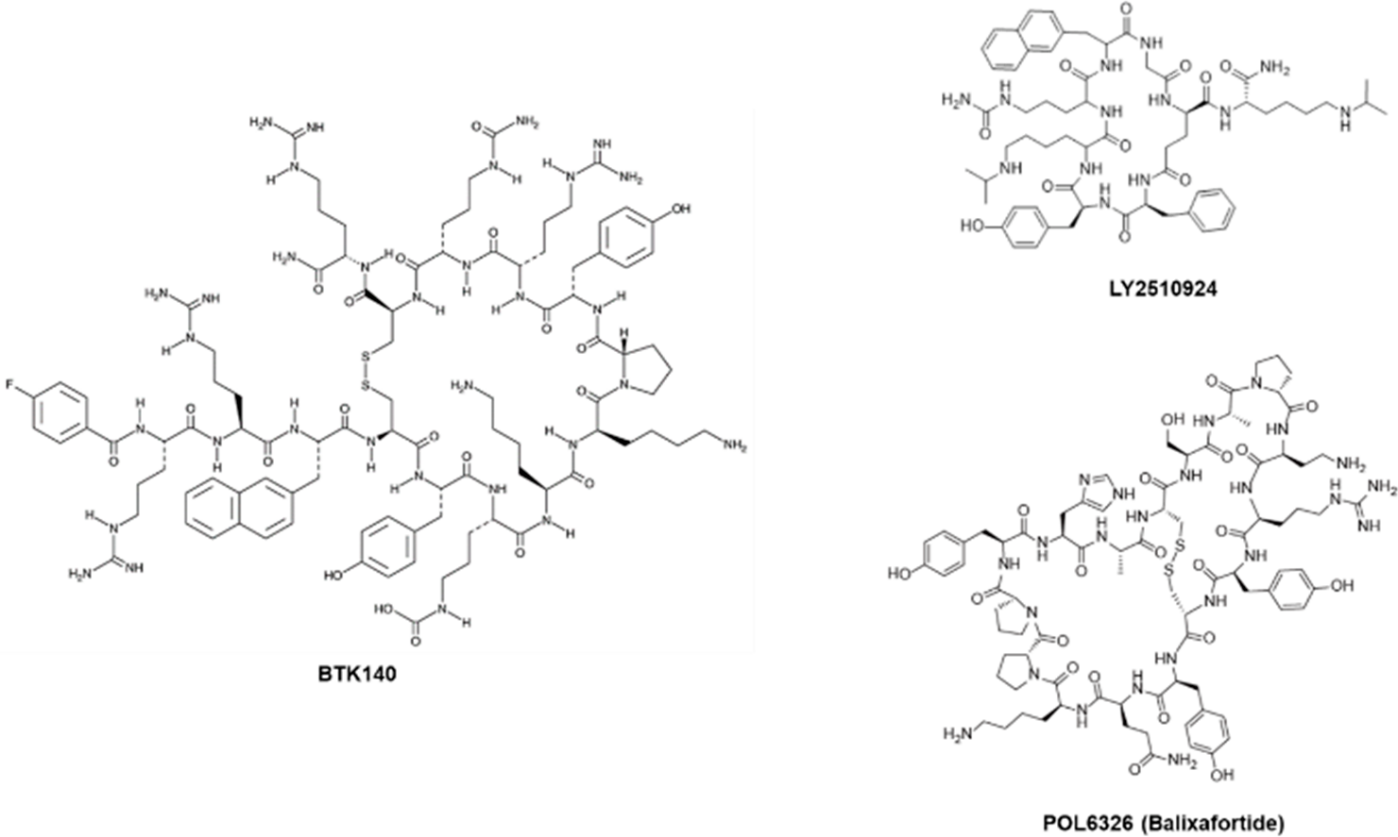
Structures of peptidic GPCR inhibitors.

Another potent and selective CXCR4 antagonist with activity in lymphoma models is LY2510924 (Figure 2), a cyclic peptide containing non-natural amino acids. It inhibited the CXCR4-mediated cell signaling in a histiocytic lymphoma U937 model in a dose-dependent manner with an IC_50_ of 0.26 nmol/L. Similarly, in NHL Namalwa cells, which also express high levels of CXCR4, the treatment with LY2510924 affected CXCR4/SDF-1-mediated cellular functions (phosphorylation of ERK and Akt) with IC_50_ values of 1.4 and 1.2 nmol/L, respectively. LY2510924 was also active against *in vivo* NHL xenograft models, in which a significant dose-dependent tumor growth reduction compared with the vehicle group and a fairly good pharmacokinetic profile were observed.^30^ Structural modeling analysis suggested that the main ligand–receptor interactions of LY2510924 are formed between its naphthalene and hydroxy benzene with the CXCR4 residues Asp^187^, Arg^188^, Gln^200^, His^113^, and Tyr^190^. Favorable interactions such as a salt bridge with Glu^288^, a H bond with Arg^30^, and a π–π stacking with Phe189 further stabilize the complex.^30^

In the past decade, multiple rounds of optimization starting from the natural product polyphemusin led to the well tolerated and highly selective next generation CXCR4 antagonist POL6326 (balixafortide) (Figure 2), a 16-amino acid cyclopeptide with one intramolecular disulfide bond. In analogy with its precursor polyphemusin, POL6326 has a β-hairpin bioactive conformation, essential for plasma stability and potency.^31,32^ The affinity of POL6326 for GPCRs was tested in a large panel of receptors, in which it showed a high 1000-fold selectivity window. Finally, another molecular strategy for the development of GPCR inhibitors is the construction of peptide–lipid conjugates, called pepducins.^33^ They are composed of a peptide sequence derived from the intracellular loops of the target GPCR, typically conjugated to palmitic acid or a lipid moiety (such as palmitate) via an amide bond.

The latter is responsible for the tethering of the pepducin into the inner leaflet of the cell membrane, whereas the peptide sequence selectively modulate the GPCR function.^34^ The bound pepducin blocks the signal transference to G protein by mimicking or stabilizing the receptor intracellular loop responsible for interactions with the G protein.^33^

Pepducins designed to target CXCR4 exhibited *in vitro* and *in vivo* efficacy in several disease models, including lymphoma. In particular, pepducins PZ-218 and PZ-210, bearing a N-terminal palmitate moiety, were designed to target the first (i1) and third (i3) intracellular loop of CXCR4. Hence, the peptide sequence of PZ-218 corresponds to MGYQKKLRSMTD, while that for PZ-210 is SKLSHSKGHQKRKALK. Both compounds inhibited CXCL12-mediated chemotaxis of Burkitt lymphoma cells (Raji and Ramos) in a dose-dependent manner, with IC_50_ values of approximately 0.3–1 μM. Furthermore, combination treatment with pepducins and rituximab significantly enhanced the cytotoxic effect of rituximab in *in vitro* and *in vivo* models. Their synergistic effect was initially evaluated in CD20-expressing Raji and Ramos lymphoma cell lines, where both PZ-218 and PZ-210 induced a 1.5–2-fold increase in the percentage of apoptotic cells compared with rituximab alone. Similarly, immunocompromised NOD/SCID mice with disseminated lymphoma showed enhanced survival compared to rituximab-treated mice. The promising efficacy of pepducins in lymphoma was further confirmed by the equivalent efficacy of PZ-218 and plerixafor in survival studies.

To assess whether the palmitate moiety and the peptide composition are essential for antagonism, pepducins without the palmitoyl portion (PZ-253 and PZ-254) and with an additional C-terminal lysine (PZ-217) were also evaluated but had no effect.^35^

These findings provide the scientific basis for the development of novel GPCR-targeted therapies for lymphoma, particularly NHL subtypes. Moreover, the combination with other chemotherapeutic agents, blocking functionally cooperative signaling pathways, may represent a new turning point in the therapeutical arsenal.

In conclusion, GPCRs have important roles in lymphocyte functions such as cell migration, proliferation, and apoptosis. Hence, genetic events that alter their expression have critical consequences in the insurgent and staging of B cell diseases, especially lymphoma. The reported results suggest that GPCRs are a potential therapeutic target in different types of this disease, which still exhibit poor survival and outcomes in response to current chemotherapy regimens. Hence, detailed knowledge of GPCR expression in malignant cells, the molecules that bind to each GPCR, and the downstream signaling pathways they activate will reveal numerous opportunities for targeted therapeutics to improve disease outcome.

From a medicinal chemistry point of view, this field of research needs to be further explored, considering that only few chemical entities have emerged so far as effective GPCR inhibitors in lymphoma models, resulting in a rather limited SAR information. In fact, besides BTK140, LY2510924, POL6326, and pepducins peptide structures, the remaining compounds can be basically divided into a family of compounds based on a cyclam structure (plerixafor, GENZ-644494) and the other incorporating heterocyclic moieties.

## Abbreviations

GPCRs: G protein-coupled receptors
FZD: frizzled receptors
SMO: smoothened receptors
PLC: phospholipase C
RhoGEF: RhoGTPase nucleotide exchange factors
ERK: extracellular regulated kinase
JNK: Janus-activated kinase
NHL: non-Hodgkin lymphoma
MCL: mantle cell lymphoma
FL: follicular lymphoma
DLBCL: diffuse large B cell lymphoma
MZL: marginal zone lymphoma
FDA: U.S. Food and Drug Administration
BM: bone marrow
ADCC: antibody-dependent cell-mediated cytotoxicity
G-CSF: granulocyte-colony stimulating factors
FD: fixed-dose
WB: weight-based
HSCs: hematopoietic stem cells
GCB: germinal center B-cell like
ABC: activated B-cell-like
BL: Burkitt’s lymphoma.

## References

[ref1] Lee Y.; Basith S.; Choi S. Recent Advances in Structure-Based Drug Design Targeting Class A G Protein-Coupled Receptors Utilizing Crystal Structures and Computational Simulations. J. Med. Chem. 2018, 61, 1–46. 10.1021/acs.jmedchem.6b01453. 28657745

[ref2] Rajagopal S.; Ponnusamy M. Overview of G-Protein Coupled Receptor. In Metabotropic GPCRs: TGR5 and P2Y Receptors in Health and Diseases; Springer: Singapore, 2018; pp 1–18.

[ref3] Yang D.; Zhou Q.; Labroska V.; Qin S.; Darbalaei S.; Wu Y.; Yuliantie E.; Xie L.; Tao H.; Cheng J.; Liu Q.; Zhao S.; Shui W.; Jiang Y.; Wang M.-W. G Protein-Coupled Receptors: Structure- and Function-Based Drug Discovery. Signal Transduct. Target. Ther. 2021, 6, 1–27. 10.1038/s41392-020-00435-w. 33414387 PMC7790836

[ref4] Swerdlow S. H.; Campo E.; Harris N. L.; Jaffe E. S.; Pileri S. A.; Stein H.; Thiele J. WHO Classification of Tumours of Haematopoietic and Lymphoid Tissues, rev. 4th ed.; IARC: Lyon, France, 2017.

[ref5] Nugent A.; Proia R. L. The Role of G Protein-Coupled Receptors in Lymphoid Malignancies. Cell. Signal. 2017, 39, 95–107. 10.1016/j.cellsig.2017.08.002. 28802842 PMC5600616

[ref6] Burger J. A.; Kipps T. J. CXCR4: A Key Receptor in the Crosstalk between Tumor Cells and Their Microenvironment. Blood 2006, 107, 1761–1767. 10.1182/blood-2005-08-3182. 16269611

[ref7] Arribas A. J.; Napoli S.; Cascione L.; Gaudio E.; Bordone-Pittau R.; Barreca M.; Sartori G.; Chiara T.; Spriano F.; Rinaldi A.; Stathis A.; Stussi G.; Rossi D.; Emanuele Z.; Bertoni F. Secondary Resistance to the PI3K Inhibitor Copanlisib in Marginal Zone Lymphoma. Eur. J. Cancer 2020, 138, S40 10.1016/S0959-8049(20)31181-3.

[ref8] Tarantelli C.; Gaudio E.; Arribas A. J.; Kwee I.; Hillmann P.; Rinaldi A.; Cascione L.; Spriano F.; Bernasconi E.; Guidetti F.; Carrassa L.; Bordone-Pittau R.; Beaufils F.; Ritschard R.; Rageot D.; Sele A.; Dossena B.; Rossi F. M.; Zucchetto A.; Taborelli M.; Gattei V.; Rossi D.; Stathis A.; Stussi G.; Broggini M.; Wymann M. P.; Wicki A.; Zucca E.; Cmiljanovic V.; Fabbro D.; Bertoni F. PQR309 Is a Novel Dual PI3K/MTOR Inhibitor with Preclinical Antitumor Activity in Lymphomas as a Single Agent and in Combination Therapy. Clin. Cancer Res. 2018, 24, 120–129. 10.1158/1078-0432.CCR-17-1041. 29066507

[ref9] Chen L.; Ouyang J.; Wienand K.; Bojarczuk K.; Hao Y.; Chapuy B.; Neuberg D.; Juszczynski P.; Lawton L. N.; Rodig S. J.; Monti S.; Shipp M. A. CXCR4 Upregulation Is an Indicator of Sensitivity to B-Cell Receptor/PI3K Blockade and a Potential Resistance Mechanism in B-Cell Receptor-Dependent Diffuse Large B-Cell Lymphomas. Haematologica 2020, 105, 1361–1368. 10.3324/haematol.2019.216218. 31471373 PMC7193488

[ref10] Ratmanova N. K.; Andreev I. A.; Trushkov I. V. Methods for the Synthesis of Immunostimulant Plerixafor. Chem. Heterocycl. Compd. 2020, 56, 30–35. 10.1007/s10593-020-02617-4.

[ref11] Ciampolini M.; Fabbrizzi L.; Perotti A.; Poggi A.; Seghi B.; Zanobini F. Dinickel and Dicopper Complexes with N,N-Linked Bis(Cyclam) Ligands. An Ideal System for the Investigation of Electrostatic Effects on the Redox Behavior of Pairs of Metal Ions. Inorg. Chem. 1987, 26, 3527–3533. 10.1021/ic00268a022.

[ref12] Yaouanc J. J.; Le Bris N.; Clément J. C.; Handel H.; des Abbayes H. ω-Mono N-Alkylation of Linear Tetraamines through the Reaction of Aldehydes and Ketones on Their Tricarbonyl Chromium, Molybdenum or Tungsten Complexes. J. Chem. Soc. Chem. Commun. 1993, 696–698. 10.1039/C39930000696.

[ref13] Filali A.; Yaouanc J. J.; Handel H. Stoichiometric Mono N-Functionalization of Tetraazamacrocycles via Phosphoryl-Protected Intermediates. Angew. Chem., Int. Ed. Engl. 1991, 30, 560–561. 10.1002/anie.199105601.

[ref14] Bernard H.; Yaouanc J. J.; Clément J. C.; des Abbayes H.; Handel H. General Route for the Synthesis of Mono N-Alkylated Derivatives of Tetraazamacrocycles. Tetrahedron Lett. 1991, 32, 639–642. 10.1016/S0040-4039(00)74848-9.

[ref15] Gardinier I.; Roignant A.; Oget N.; Bernard H.; Yaouanc J. J.; Handel H. Trivalent Protecting Groups for the Synthesis of Symmetrical and Unsymmetrical Bis-Tetraazamacrocycles. Tetrahedron Lett. 1996, 37, 7711–7714. 10.1016/0040-4039(96)01722-4.

[ref16] Reinholdt L.; Laursen M. B.; Schmitz A.; Bødker J. S.; Jakobsen L. H.; Bøgsted M.; Johnsen H. E.; Dybkaer K. The CXCR4 Antagonist Plerixafor Enhances the Effect of Rituximab in Diffuse Large B-Cell Lymphoma Cell Lines. Biomark. Res. 2016, 4, 1–12. 10.1186/s40364-016-0067-2. 27307990 PMC4908729

[ref17] Hu Y.; Gale M.; Shields J.; Garron C.; Swistak M.; Nguyen T. H.; Jacques G.; Fogle R.; Siders W.; Kaplan J. Enhancement of the Anti-Tumor Activity of Therapeutic Monoclonal Antibodies by CXCR4 Antagonists. Leuk. Lymphoma 2012, 53, 130–138. 10.3109/10428194.2011.601698. 21740294

[ref18] Bodart V.; Anastassov V.; Darkes M. C.; Idzan S. R.; Labrecque J.; Lau G.; Mosi R. M.; Neff K. S.; Nelson K. L.; Ruzek M. C.; Patel K.; Santucci Z.; Scarborough R.; Wong R. S. Y.; Bridger G. J.; MacFarland R. T.; Fricker S. P. Pharmacology of AMD3465: A Small Molecule Antagonist of the Chemokine Receptor CXCR4. Biochem. Pharmacol. 2009, 78, 993–1000. 10.1016/j.bcp.2009.06.010. 19540208

[ref19] Rosenkilde M. M.; Gerlach L. O.; Jakobsen J. S.; Skerlj R. T.; Bridger G. J.; Schwartz T. W. Molecular Mechanism of AMD3100 Antagonism in the CXCR4 Receptor: Transfer of Binding Site to the CXCR3 Receptor. J. Biol. Chem. 2004, 279, 3033–3041. 10.1074/jbc.M309546200. 14585837

[ref20] Gerlach L. O.; Skerlj R. T.; Bridger G. J.; Schwartz T. W. Molecular Interactions of Cyclam and Bicyclam Non-Peptide Antagonists with the CXCR4 Chemokine Receptor. J. Biol. Chem. 2001, 276, 14153–14160. 10.1074/jbc.M010429200. 11154697

[ref21] Rosenkilde M. M.; Gerlach L. O.; Hatse S.; Skerlj R. T.; Schols D.; Bridger G. J.; Schwartz T. W. Molecular Mechanism of Action of Monocyclam versus Bicyclam Non-Peptide Antagonists in the CXCR4 Chemokine Receptor. J. Biol. Chem. 2007, 282, 27354–27365. 10.1074/jbc.M704739200. 17599916

[ref22] Debnath B.; Xu S.; Grande F.; Garofalo A.; Neamati N. Small Molecule Inhibitors of CXCR4. Theranostics 2013, 3, 47–75. 10.7150/thno.5376. 23382786 PMC3563081

[ref23] Skerlj R. T.; Bridger G. J.; Kaller A. I.; McEachern E. J.; Crawford J. B.; Zhou Y.; Atsma B.; Langille J.; Nan S.; Veale D.; Wilson T.; Harwig C.; Hatse S.; Princen K.; De Clercq E.; Schols D. Discovery of Novel Small Molecule Orally Bioavailable C-X-C Chemokine Receptor 4 Antagonists That Are Potent Inhibitors of T-Tropic (X4) HIV-1 Replication. J. Med. Chem. 2010, 53, 3376–3388. 10.1021/jm100073m. 20297846

[ref24] Pansy K.; Feichtinger J.; Ehall B.; Uhl B.; Sedej M.; Roula D.; Pursche B.; Wolf A.; Zoidl M.; Steinbauer E.; Gruber V.; Greinix H. T.; Prochazka K. T.; Thallinger G. G.; Heinemann A.; Beham-Schmid C.; Neumeister P.; Wrodnigg T. M.; Fechter K.; Deutsch A. J. The CXCR4-CXCL12-Axis Is of Prognostic Relevance in DLBCL and Its Antagonists Exert pro-Apoptotic Effects in Vitro. Int. J. Mol. Sci. 2019, 20, 4740 10.3390/ijms20194740. 31554271 PMC6801866

[ref25] Ros-Blanco L.; Anido J.; Bosser R.; Esté J.; Clotet B.; Kosoy A.; Ruíz-Ávila L.; Teixidó J.; Seoane J.; Borrell J. I. Noncyclam Tetraamines Inhibit CXC Chemokine Receptor Type 4 and Target Glioma-Initiating Cells. J. Med. Chem. 2012, 55, 7560–7570. 10.1021/jm300862u. 22909088

[ref26] Recasens-Zorzo C.; Cardesa-Salzmann T.; Petazzi P.; Ros-Blanco L.; Esteve-Arenys A.; Clot G.; Guerrero-Hernández M.; Rodríguez V.; Soldini D.; Valera A.; Moros A.; Climent F.; González-Barca E.; Mercadal S.; Arenillas L.; Calvo X.; Mate J. L.; Gutiérrez-García G.; Casanova I.; Mangues R.; Sanjuan-Pla A.; Bueno C.; Menéndez P.; Martínez A.; Colomer D.; Tejedor R. E.; Teixidó J.; Campo E.; López-Guillermo A.; Borrell J. I.; Colomo L.; Pérez-Galán P.; Roué G. Pharmacological Modulation of CXCR4 Cooperates with BET Bromodomain Inhibition in Diffuse Large B-Cell Lymphoma. Haematologica 2019, 104, 778–788. 10.3324/haematol.2017.180505. 29954928 PMC6442946

[ref27] Peled A.; Abraham M.; Avivi I.; Rowe J. M.; Beider K.; Wald H.; Tiomkin L.; Ribakovsky L.; Riback Y.; Ramati Y.; Aviel S.; Galun E.; Shaw H. L.; Eizenberg O.; Hardan I.; Shimoni A.; Nagler A. The High-Affinity CXCR4 Antagonist BKT140 Is Safe and Induces a Robust Mobilization of Human CD34+ Cells in Patients with Multiple Myeloma. Clin. Cancer Res. 2014, 20, 469–479. 10.1158/1078-0432.CCR-13-1302. 24246358

[ref28] Chen J.; Xu-Monette Z. Y.; Deng L.; Shen Q.; Manyam G. C.; Martinez-Lopez A.; Zhang L.; Montes-Moreno S.; Visco C.; Tzankov A.; Yin L.; Dybkaer K.; Chiu A.; Orazi A.; Zu Y.; Bhagat G.; Richards K. L.; Hsi E. D.; Choi W. W. L.; van Krieken J. H.; Huh J.; Ponzoni M.; Ferreri A. J. M.; Zhao X.; Møller M. B.; Farnen J. P.; Winter J. N.; Piris M. A.; Pham L.; Young K. H. Dysregulated CXCR4 Expression Promotes Lymphoma Cell Survival and Independently Predicts Disease Progression in Germinal Center B-Cell-like Diffuse Large B-Cell Lymphoma. Oncotarget 2015, 6, 5597–5614. 10.18632/oncotarget.3343. 25704881 PMC4467389

[ref29] Beider K.; Ribakovsky E.; Abraham M.; Wald H.; Weiss L.; Rosenberg E.; Galun E.; Avigdor A.; Eizenberg O.; Peled A.; Nagler A. Targeting the CD20 and CXCR4 Pathways in Non-Hodgkin Lymphoma with Rituximab and High-Affinity CXCR4 Antagonist BKT140. Clin. Cancer Res. 2013, 19, 3495–3507. 10.1158/1078-0432.CCR-12-3015. 23637121

[ref30] Peng S. B.; Zhang X.; Paul D.; Kays L. M.; Gough W.; Stewart J.; Uhlik M. T.; Chen Q.; Hui Y. H.; Zamek-Gliszczynski M. J.; Wijsman J. A.; Credille K. M.; Yan L. Z. Identification of LY2510924, a Novel Cyclic Peptide CXCR4 Antagonist That Exhibits Antitumor Activities in Solid Tumor and Breast Cancer Metastatic Models. Mol. Cancer Ther. 2015, 14, 480–490. 10.1158/1535-7163.MCT-14-0850. 25504752

[ref31] Batur G.; Ermert P.; Zimmermann J.; Obrecht D. Macrocycle Therapeutics to Treat Life-Threatening Diseases. Chimia (Aarau). 2021, 75, 508–513. 10.2533/chimia.2021.508. 34233814

[ref32] Zimmermann J.; Remus T.; Lemercier G.; Barker D.; Obrecht D.; Gambino G.; Douglas G. Anti-Tumor Cell Activity and in Vitro Profile of the next Generation CXCR4 Antagonist Balixafortide. Ann. Oncol. 2018, 29, viii103 10.1093/annonc/mdy272.312.

[ref33] Covic L.; Gresser A. L.; Talavera J.; Swift S.; Kuliopulos A. Activation and Inhibition of G Protein-Coupled Receptors by Cell-Penetrating Membrane-Tethered Peptides. Proc. Natl. Acad. Sci. U. S. A. 2002, 99, 643–648. 10.1073/pnas.022460899. 11805322 PMC117359

[ref34] Adlere I.; Caspar B.; Arimont M.; Dekkers S.; Visser K.; Stuijt J.; de Graaf C.; Stocks M.; Kellam B.; Briddon S.; Wijtmans M.; de Esch I.; Hill S.; Leurs R. Modulators of CXCR4 and CXCR7/AckR3 Function. Mol. Pharmacol. 2019, 96, 737–752. 10.1124/mol.119.117663. 31548340

[ref35] O’Callaghan K.; Lee L.; Nguyen N.; Hsieh M. Y.; Kaneider N. C.; Klein A. K.; Sprague K.; Van Etten R. A.; Kuliopulos A.; Covic L. Targeting CXCR4 with Cell-Penetrating Pepducins in Lymphoma and Lymphocytic Leukemia. Blood 2012, 119, 1717–1725. 10.1182/blood-2011-04-347518. 22186993 PMC3286348

